# Spinal Involvement in Patients with Chronic Non-Bacterial Osteomyelitis (CNO): An Analysis of Distinctive Imaging Features

**DOI:** 10.3390/jcm12237419

**Published:** 2023-11-30

**Authors:** Marta Byrdy-Daca, Marek Duczkowski, Iwona Sudoł-Szopińska, Marta Żelewska, Krzysztof Piłat, Filip Daca, Michał Nieciecki, Paweł Sztwiertnia, Jerzy Walecki, Andrzej Cieszanowski, Jan Świątkowski, Marlena Bereźniak, Katarzyna Sułkowska, Jarosław Czubak, Marek Gołębiowski, Piotr Palczewski

**Affiliations:** 1 I Department of Clinical Radiology, Medical University of Warsaw, 02-091 Warsaw, Poland; diagnostyka.daca@gmail.com (M.B.-D.); krzysztof.pilat@uckwum.pl (K.P.); jan.swiatkowski@uckwum.pl (J.Ś.); marlena.berezniak@gmail.com (M.B.); katarzyna.sulkowska@uckwum.pl (K.S.); marek.golebiowski@wum.edu.pl (M.G.); 2 Department of Diagnostic Imaging, Institute of Mother and Child, 01-211 Warsaw, Poland; marek.duczkowski@imid.med.pl; 3 Department of Radiology, The National Institute of Geriatrics, Rheumatology and Rehabilitation, 02-637 Warsaw, Poland; iwona.sudol-szopinska@spartanska.pl (I.S.-S.); michal.nieciecki@spartanska.pl (M.N.); 4 Gruca Orthopaedic and Trauma Teaching Hospital, Center of Postgraduate Medical Education, 05-400 Otwock, Poland; martazelewska1@gmail.com (M.Ż.); radiologiacmkp@spskgruca.pl (P.S.); rtg@cskmswia.gov.pl (J.W.); kl.ort.dz@spskgruca.pl (J.C.); 5 Independent Researcher, 00-195 Warszawa, Poland; filip.hubert.daca@gmail.com; 6 The Maria Skłodowska Curie Memorial Cancer Centre and Institute of Oncology, 05-077 Warsaw, Poland; radiologia1@pib-nio.pl

**Keywords:** CRMO, SAPHO, chronic nonbacterial osteomyelitis, imaging

## Abstract

Spinal involvement by chronic non-bacterial osteomyelitis (CNO) has been increasingly reported in recent years, often being presented as a diagnostic dilemma requiring differential diagnosis with bacterial spondylodiscitis and/or neoplasia. This study was aimed at identifying the imaging features of CNO facilitating its differentiation from other spinal diseases. Two radiologists assessed the imaging studies of 45 patients (16 male and 29 female, aged from 6 to 75 years, 15 children) with CNO collected from 5 referential centers. Spinal lesions were found in 17 patients (2 children and 15 adults), most often in the thoracic spine. In children, the lesions involved short segments with a destruction of vertebral bodies. In adults, the main findings were prominent bone marrow edema and osteosclerosis, endplate irregularities, and ankylosing lesions extending over long segments; paraspinal inflammation was mild and abscesses were not observed. In both children and adults, the involvement of posterior elements (costovertebral and facet joints) emerged as an important discriminator between CNO and neoplasia/other inflammatory conditions. In conclusion, a careful inspection of imaging studies may help to reduce the number of biopsies performed in the diagnostic process of CNO.

## 1. Introduction

Chronic non-bacterial osteomyelitis (CNO) is an auto-inflammatory disease combining sterile, usually multifocal, osteomyelitis with arthritis, enthesitis, skin abnormalities, and less frequently other autoimmune disorders [1,2]. It is believed that CNO is induced by *Propionibacterium acnes* infection, which in genetically predisposed individuals provokes an impaired immune reaction with overproduction of interleukins and TNF-α [3]. The terminology pertaining to CNO is heterogenous, reflecting the variability of clinical and imaging manifestations; however, two disease phenotypes are most often distinguished: CRMO (chronic recurrent multifocal osteomyelitis) and SAPHO (synovitis, acne, pustulosis, hyperostosis, osteitis) syndrome [1,4]. CRMO typically presents in children (peaking at 10 years of age) and most often involves the clavicle and metaphyses/diaphyses of the long bones, while SAPHO is diagnosed in adults (most often between 30–50 years of age) and a classic location is the anterior thoracic wall (ATW), yet both phenotypes or their variants may appear both in children and adults [1,2,4,5,6,7,8]. CRMO and SAPHO are considered rare diseases; however, there are wide discrepancies in reported incidence, ranging from 1:10,000 to 1–2:1,000,000 [1,5,9,10,11]. Those may be partly attributable to interpopulational genetic differences, but it has also been suggested that a true incidence of CNO may be underestimated [6,12].

In recent years, with a growing knowledge of CNO manifestations, especially on diagnostic imaging, more cases are being diagnosed and the attention has been brought to the involvement of axial skeleton [13,14]. A recent study on a cohort of 345 patients with SAPHO syndrome, showed that the involvement of axial skeleton (spine and sacroiliac joints) is common (55.6% of patients) and related to a distinct clinical course [15]. As many as 26% of patients with CRMO are reported to have spinal involvement [16]. Considering that spinal and chest imaging are one of the most commonly performed investigations, an incidental detection of CNO lesions involving the spine, may pose a diagnostic challenge requiring a differential diagnosis with other more common spinal diseases, especially bacterial spondylodiscitis and neoplastic disease [17,18]. As recent reports show, the diagnosis of CNO is still difficult, often being delayed for months or even years from clinical presentation or initial detection of bone lesions, with multiple imaging studies and invasive procedures being performed underway [5,9,18]. The aim of this study was to perform a detailed analysis of the imaging features of spinal involvement in a group of 45 patients diagnosed with CNO and to compare them with those described in the literature in order to identify features facilitating differentiation from other diseases involving the spinal column. The identification of such features would possibly allow for simplifying a diagnostic algorithm and reducing the number of both imaging studies and biopsies performed on the way to the correct diagnosis.

## 2. Materials and Methods

This retrospective study was approved by the institutional review board (decision no. AKBE 197/2022) with a waiver of informed patient consent.

The study group consisted of 45 patients (16 male and 29 female, aged from 6 to 75 years, 15 children) with CRMO and SAPHO syndrome. The diagnoses were based on the Bristol criteria in the CRMO group and the Kahn criteria in the SAPHO group [19,20] (Table 1). Bone biopsy was performed on 19 patients. Bacterial, viral, and fungal cultures and stains were negative.

**Figure 1:**
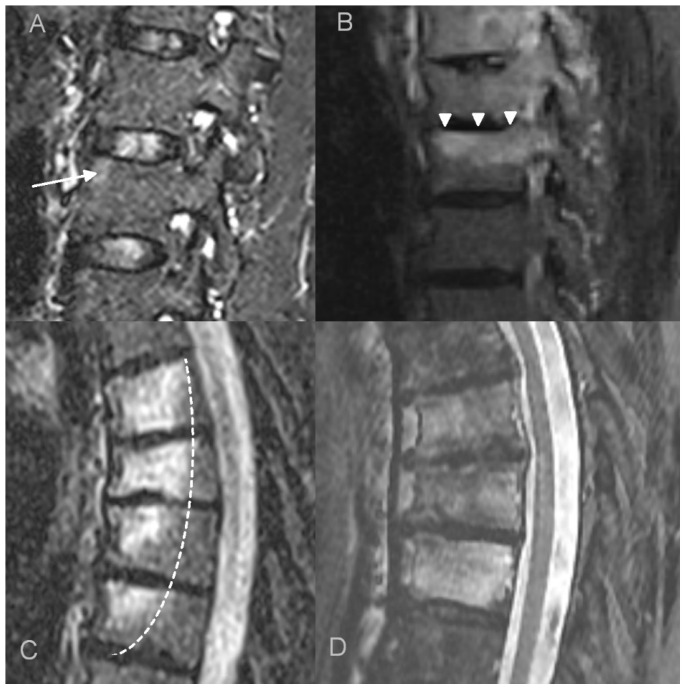
Bone marrow edema (BME) patterns appreciated on T2 FS/STIR MRI images. (**A**). corner inflammatory lesions (arrow); (**B**). propagating (arrowheads); (**C**). semicircular/curvilinear (dotted line); (**D**). diffuse—patchy (in the upper two vertebrae) and solid (in the lowest vertebra).

**Figure 2:**
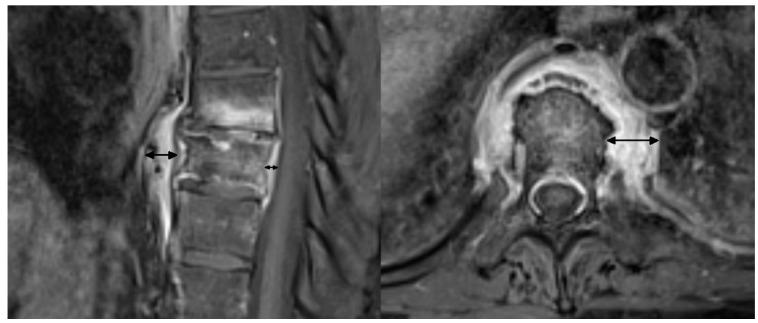
Measurement technique of the thickness of paraspinal inflammation (double-headed arrows) on sagittal and axial T1 fat saturated contrast enhanced images.

Two radiologists (M.B-D. and P.P.) performed a consensus reading of all spinal imaging studies provided in the DICOM format by the five participating centers. The following features were assessed:

1. the number and location of affected vertebrae and continuity or non-continuity of involvement; continuous involvement was defined as encompassing at least two spinal motion segments (i.e., ≥3 vertebrae),
2. bone marrow edema (BME) presence and pattern (Figure 1):
  - vertebral body corner inflammatory lesions (CIL)—active Romanus lesion defined as small foci of BME at the vertebral corners,
  - propagating BME (larger lesions extending along the endplates),
  - semicircular/curvilinear pattern of BME involving the anterior vertebral bodies as described by Peffers et al. and MCGauvran et al. [21,22],
  - diffuse solid or patchy BME involving the most of/whole vertebral body,
3. the features of intervertebral disk disease:
  e. general features of disc disease on plain films, computed tomography (CT) and magnetic resonance imaging (MRI): disc height reduction, endplate erosions, subchondral sclerosis, and intervertebral fusion,
  f. active aseptic spondylodiscitis (inflammatory Anderson lesion) on MRI defined as high disk signal on T2 with or without disc enhancement,
  g. unstable Anderson lesion defined as a pseudoarthrosis in an ankylotic spinal region,
4. paraspinal soft-tissue abnormalities:
  h. the thickness of paravertebral soft tissue inflammation measured on both sagittal and axial T2 fat saturated or T1 fat saturated contrast enhanced images and the thickness of intracanal epidural inflammation measured on sagittal images (Figure 2),
  i. presence of fluid collections/abscesses,
5. osteodestructive lesions and secondary vertebral deformities with pathologic fractures defined as >20% vertebral collapse with no history of trauma; smaller deformities were classified either as wedge, biconcave or crush,
6. presence and grade of a secondary spinal canal stenosis: in the lumbar spine assessed according to Park criteria and in both the cervical and thoracic spine according to Kang criteria [23,24],
7. osteosclerotic lesions defined as smaller foci of sclerosis adjacent to vertebral body walls or diffuse sclerosis (ivory vertebra),
8. paravertebral ossification: presence of osteophytes (marginal or non-marginal) and syndesmophytes, and number of fused segments,
9. facet joint arthropathy,
10. costovertebral joint arthropathy.

In patients with imaging studies performed at more than one time point, the dynamics of the spinal lesions were assessed. The length of imaging follow up (defined as the period between the first and last spinal imaging study available) was recorded and the intensity of abnormalities was compared between the studies and classified either as: stable, progression, or regression.

## 3. Results

Spinal involvement was present in 17 patients from the study group (38%): two children (13%, 1 male and 1 female) and fifteen adults (50%, 4 male and 11 female). The average age at the initial study in adults was 53 years and in children was 10.5 years. Bone biopsy was performed in six patients with spinal involvement: vertebral biopsy in five patients and sternal biopsy in one patient. In one patient, a 56-year-old woman, the diagnosis of CNO was preceded by a two year history of a recurring palmoplantar pustulosis; in other patients with spinal involvement skin lesions were not observed. Detailed demographic data of patients with spinal involvement, diagnostic studies they underwent, distribution of lesions, and duration of follow up are presented in Table 2.

The thoracic spine was most commonly involved (*n* = 16), followed by the lumbar (*n* = 4) and cervical spine (*n* = 1); four patients had lesions in more than one spinal region. Only one patient had lesions outside of the thoracic spine (in the lumbar spine) without a concomitant thoracic involvement. In adults, spinal involvement was most often accompanied by lesions in the anterior thoracic wall (13 patients, 86.7%), while both pediatric patients had a concomitant long bone involvement.

The total number of involved vertebrae was 7.76 per patient (min 1, max 15). Continuous involvement of multiple spinal segments was present in sixteen patients with concomitant non-continuous changes in four patients. In one patient with a single vertebra involved in the initial study, a progression to a continuous involvement of three vertebrae was observed during follow up.

Bone marrow edema (BME) was present in all 12 patients who underwent MRI. Different patterns of BME coexisted: in most patients, diffuse vertebral body BME was seen (67%), followed by BME propagating along the endplates (50%) and CIL (40%). Semicircular pattern of BME was observed in three patients (25%), However, it involved the highest number of vertebrae (Table 3).

Osteodestructive lesions were most pronounced in pediatric patients leading to pathologic fractures in both of them (two fractures in each patient with subsequent vertebra plana deformity). In one adult patient (female, aged 39), deep erosions along the endplates were observed. In other adults, smaller endplate erosions were noted, often more prevalent in anterior parts of the vertebral bodies with wedge deformity seen in seven patients (mean 1.57 vertebral body per patient) at the top of kyphosis. In one patient with an isolated lumbar spine involvement, a biconcave deformity of L5 vertebral body was observed.

Osteosclerotic lesions appeared in all patients who underwent CT or plain film. The location was overlapping with BME; ivory vertebrae were noted in five patients (mean 2.4 vertebrae per patient).

The features of intervertebral disc disease (disc height reduction, endplate erosions, subchondral sclerosis, or intervertebral fusion) were common and occurred in all patients who underwent CT or X-rays on an average of six disc levels per patient. However, active aseptic spondylodiscitis (inflammatory Anderson lesion) was demonstrated by MRI only in five patients (none of the children) on an average of two disc levels per patient.

Paraspinal soft tissue inflammation occurred in 10 adult patients and in none of the children. The thickness of prespinal inflammation ranged from 5 to 21 mm on axial images (mean 10.8 mm) and from 2.7 to 13 mm (mean 7.3 mm) on sagittal images, while the thickness of epidural inflammation ranged from 0.8 to 8.9 mm (mean 3.7 mm). Paraspinal abscesses were not observed.

Secondary spinal canal stenosis was observed in both children due to kyphotic deformity and in seven adults due to epidural inflammation; the stenosis was mild to moderate (grade 1 in six patients and grade 2 in three patients, according to Kang criteria).

Ankylosing lesions were common in patients older than 39 years. Syndesmophytes occurred in 14 patients and involved longer, continuous parts of the spine with an average of 7.2 disc levels involved per patient. Acquired anterior block vertebrae occurred in 14 patients, on average at five disc levels per patient. The involvement of the posterior elements was common both in pediatric and adult patients with an average of 11.9 facet joints and 10.6 costovertebral joints involved per patient. The ankylosis of facet and costovertebral joints was observed only in adult patients, respectively in seven (mean 9 joints per patient) and in five patients (mean 11.2 joints per patient).

The features of an unstable Anderson lesion were detected in three patients. In two patients (65-year-old male and 68-year-old female), the lesions were detected incidentally on CT performed for suspected SARS-CoV-2 infection and pulmonary embolism. In one patient, a 54-year-old female, the pseudoarthrosis appeared during a follow-up, 2 years after the initial study, and was associated with an aggravation of pain. All pseudoarthroses were found in the thoracic region (Th4/Th5, Th9/Th10, and Th10/Th11 disc levels).

Eleven patients underwent multiple imaging studies (both children and nine adults). The average follow up lasted 45 months (1 month–192 months, median 37.5 months). In both children, a follow-up demonstrated a progression of destruction of vertebral bodies and kyphosis, eventually requiring surgery with posterior fusion. In adults, the most striking feature was a long persistence of BME that was present through the whole length of follow up. In six adults with a progressive involvement of a longer spinal segment, the greatest dynamics of progression were observed in the first two years of follow-up, after which the lesions stabilized in some patients with a minimal regression of BME (being replaced by osteosclerosis on plain film/CT). In four adults, a progressive spinal ankylosis with a formation of new syndesmophytes was observed.

## 4. Discussion

The aim of this study was to analyze imaging features of spinal involvement in CNO and identify those facilitating a differential diagnosis with other diseases. Based on our results and the literature review, we made two main observations. First, the features of CNO differ in children/young adults and older patients, demanding a different differential diagnosis. Second, in both children and adults, it is possible to identify imaging features that in combination are highly specific for the diagnosis of CNO.

Similarly to previous reports, the inflammatory lesions in patients with CNO showed a predilection to the thoracic spine [13,14,22,25,26]. In adults, the lesions were most often multilevel and continuous, while in children, shorter segments were involved, which is also consistent with previous reports [15,22,25,26]. The main difference between younger and older patients was the dominance of osteodestructive lesions in the former and of enthesitis and arthritis with progressive ankylosis in the latter.

**Figure 3:**
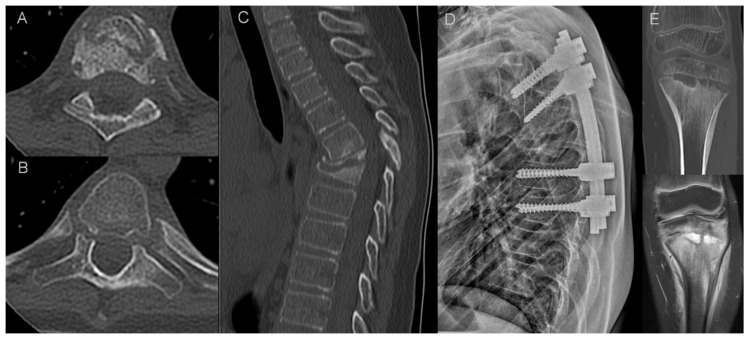
Imaging findings in a 10-year-old patient with CRMO (patient 16.). Axial CT scans show erosions in the facet (**A**) and costovertebral (**B**) joints. Sagittal CT reformat shows osteodestructive lesions with vertebra plana deformity (**C**) that led to progressive kyphosis with spinal stenosis requiring transpedicular stabilization (**D**). Another site of the disease was found in the tibia (**E**) with CT and MRI showing metaphyseal osteolysis with adjacent osteosclerosis, extensive bone marrow edema and solid periosteal reaction.

In two pediatric patients of our cohort, there was a destruction of vertebral bodies with vertebra plana deformity and progressive kyphosis that eventually required surgery (Figure 3). There are several reports on an aggressive course of CRMO in the pediatric population with a rapid destruction of vertebral bodies and vertebra plana formation in some patients, on multiple levels, as recapped in the article by Anderson and coworkers [25]. This aggressive course requires differential diagnosis with pyogenic spondylodiscitis and neoplastic disease [26]. To make things more difficult, a clinical presentation of pediatric bacterial spondylodiscitis may be similar to CRMO, since laboratory findings are often unremarkable, showing only a slight to moderate increase in inflammatory markers [27]. The hallmarks of pyogenic spondylodiscitis are disc signal changes and paraspinal soft tissue inflammation with phlegmon or abscess formation [27,28]. Those features were completely absent in both children with vertebra plana and one young adult with marginal vertebral body destructive lesions in our study group. Langerhans cell histiocytosis (LCH) is considered the most common cause of vertebra plana in children; however, a recent report has showed a higher-than-expected incidence of non-LCH diagnoses with a high rate of other malignancies and non-malignant causes, including CRMO [29,30,31]. The lack of vertebral wall bulging and paraspinal soft tissue mass may be helpful in differentiating CRMO from malignancy. However, in our opinion, the features of inflammatory arthritis of costovertebral and facet joints are the most important discriminator. Erosions in costovertebral and facet joints were present in all patients with vertebral body destruction in our study group (Figure 3). According to our experience, those subtle changes in spinal joints are easier to appreciate on CT than MRI thanks to higher resolution and multiplanar reformatting capabilities of the former. In addition, the distribution of lesions may help in differentiating CRMO from LCH, which most often presents as a monofocal osseous lesion [29,30]. Both children in our material had a concomitant long bone involvement (Figure 3).

**Figure 4:**
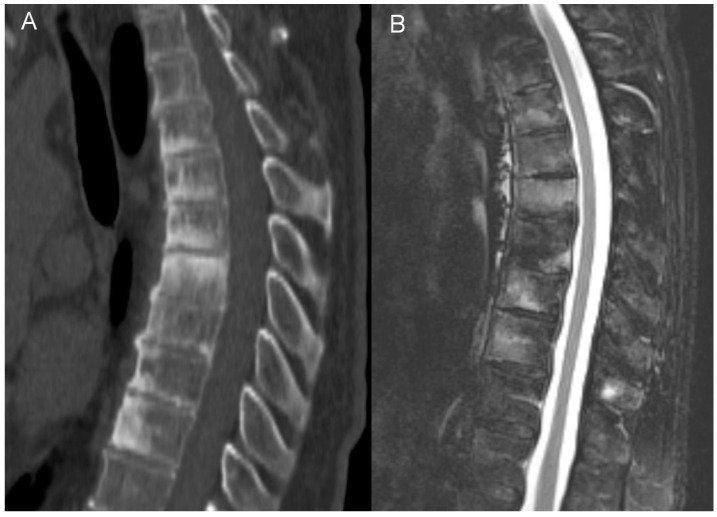
Imaging findings of the thoracic spine involvement in a 65-year-old patient with SAPHO syndrome. CT (**A**) shows multilevel, contiguous vertebral osteosclerosis, endplate erosions, and early syndesmophyte formation. MRI (**B**) shows diffuse and propagating patterns of BME and mild prevertebral soft tissue inflammation.

In adults, the main findings were BME, osteosclerosis, endplate irregularities, and a spectrum of ankylosing lesions. Similar to previous publications, the lesions were multilevel and contiguous [21,22,32,33]. Curvilinear/semicircular pattern of BME found in the majority of patients in the study by McGvuran and coworkers was less common in our material three patients); however, both BME and osteosclerosis overlapping BME on plain film/CT were definitely the most striking abnormalities (Figure 4) [22]. We also observed a long persistence of BME that was present through the whole length of follow up, even in patients followed for more than 10 years. On MRI, contrast enhancement was observed in the areas of BME, but also in the linear fashion along the endplates and frequently in the non-ankylosed costovertebral and facet joints. Paraspinal soft tissue involvement was observed in 10 out of 12 patients subjected to MRI (83%), more often than in previous publications [22,34]. In agreement with previous reports, most of the intervertebral discs showed decreased T2 signal corresponding to degeneration rather than the high signal seen in discitis [22]. Vertebral deformities were limited to anterior wedging, most pronounced at the apex of thoracic kyphosis. Ankylosing lesions were prevalent and, as previously reported, showed a progressive character on follow up [35,36]. Anterior bony bridging resembling this observed in diffuse idiopathic skeletal hyperostosis (DISH) was accompanied by intervertebral fusion and facet joint ankylosis. Contrary to previous reports, we have not observed the resolution of hyperostosis once ankylosis was complete [36].

Considering the above mentioned imaging features of CNO, the main differential diagnoses in adults include spinal tuberculosis (TB), spondyloarthropathies, mainly ankylosing spondylitis (AS), and DISH. Tuberculous spondylitis is the most common form of extrapulmonary tuberculosis and may show several similarities with CNO, both in terms of insidious clinical course and imaging findings [37,38,39]. Preferential thoracic involvement, contiguous spread to several vertebral levels along the anterior ligamentous structures with anterior vertebral scalloping, and relative sparing of intervertebral discs are considered classic for TB and may produce images resembling SAPHO [37,38]. While extensive reactive sclerosis is not typical for TB, cases with ivory vertebra appearance have been reported [40]. Both TB and CNO may lead to vertebral body destruction; however, in CNO it is rarely severe [13,14,37,38]. The most typical feature of TB are paraspinal abscesses containing calcifications, present in approximately 70% of cases [41]. None of our patients had calcifications nor fluid collections within the paraspinal soft tissues. In addition, TB rarely involves the posterior elements, which is opposite to our patients who presented with multilevel abnormalities at this site [37,38]. Paraspinal soft tissue abnormalities are also important in differential diagnosis with AS and DISH, in which they are absent [42,43]. Also, BME in AS is typically not as extensive as in CNO and is associated with osteopenia instead of osteosclerosis [44,45]. Moreover, vertebral involvement in AS is usually continuous and skip lesions are very rare [46,47,48]. In DISH, osteosclerosis is also absent (apparently increased bone density on plain film being due to overlapping paraspinal ossifications), BME is seldom seen and usually limited to small vertebral body corner lesions, and facet joints are not involved [40,49,50]. Psoriatic arthritis may produce findings very similar to SAPHO; however, differentiation between those two entities is of less importance, since researchers generally agree that there is a significant overlap between the two, both clinically and radiologically (even if there is still some resistance to considering them a part of the disease spectrum) [20,51,52,53].

**Figure 5:**
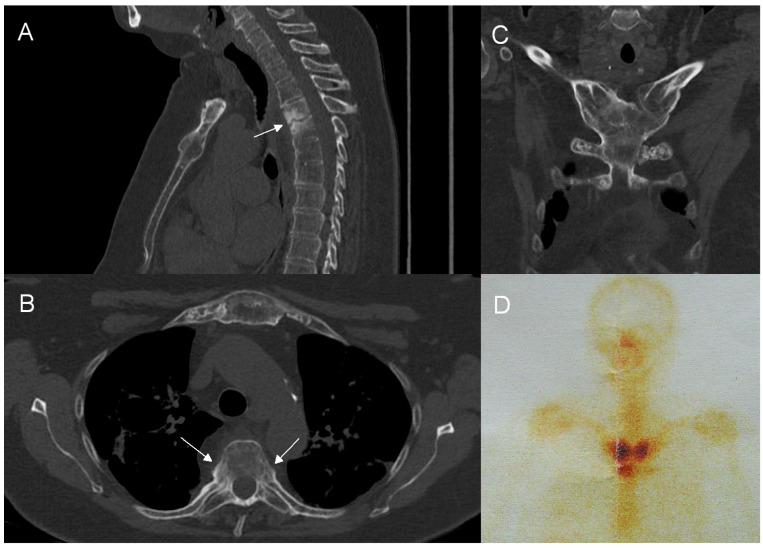
Concomitant involvement of the anterior chest wall and spine in a 54-year-old patient with SAPHO syndrome. CT shows the involvement of the thoracic spine (**A**) with an unstable Andersson lesion (arrow) and costovertebral joints ankylosis (arrows, (**B**)) and the hyperostosis and ankylosis of sternoclavicular joints, I costovertebral joints, and manubriosternal junction (**C**). An enlarged image from whole-body bone scintigraphy (**D**) shows an increased radiotracer uptake around sternoclavicular joints (‘bull’s head’ sign).

While the combination of pronounced BME and osteosclerosis, costovertebral and facet joint involvement, and limited paraspinal inflammation without abscess seems highly specific for CNO (Table 4), it is imaginable that in a given patient not all features may be present. Multifocality is a recognized feature of CNO, included both in Bristol and Khan criteria [19,20]. Accordingly, our results show that in equivocal cases, search for other sites of involvement should precede invasive procedures. In adults, the anterior chest wall is the most common site affected by CNO (in 65–90% of cases) [13,14,54]. In our material, ATW was involved in 13 out of 15 adults (86.7%) (Table 1) (Figure 5). While ATW may be affected by axial spondyloarthritis and DISH, infectious arthritis in this area is extraordinarily rare [37,55,56,57]. Therefore, when performing CT of the thoracic spine, it is of note that this additional diagnostic information potentially narrowing the differential diagnosis may be easily obtained by enlarging the field of view to include ATW, provided that the raw study data are still available on the technologist console. It is worth underlining that a retrospective assessment of patients’ medical data revealed that in our material among six biopsies performed, three biopsies taken in the early phase of the diagnostic process of spinal lesions might have been avoided, since all three patients had a concomitant ATW involvement (Table 2). In children, clavicle and long bones, especially the tibia, are most often affected [13,19].

Whole-body bone scintigraphy (WBBS), single-photon emission computed tomography (SPECT/CT), and F-18 fluorodeoxyglucose-positron emission tomography/computed tomography (18F-FDG PET/CT) are all capable of demonstrating multiple sites of involvement in CNO, including the characteristic “bull-head” sign resulting from ATW involvement [58,59] (Figure 5). An 18F-FDG PET scan shows moderate to substantial agreement with WBBS and CT in revealing lesions in the spine and ATW; however, the radiation dose is substantially higher [58,60]. Recently, whole-body MRI has proved no less effective than nuclear medicine in defining the extent of CNO in both adult and pediatric patients, thus being helpful in advancing the diagnosis in equivocal cases without an exposure to ionizing radiation [61,62,63,64]. The role of imaging seems to grow in importance as more authors point to the often asymptomatic course of CNO bone lesions and to the inconstant presence of skin lesions, once thought to constitute a classic component of the syndrome [63]. Now, it is estimated that skin changes, such as palmoplantar pustulosis or severe acne, are present only in approximately 58% of adults and in 23–80% of children/adolescents with CNO [13,54]. In a recent study by Okuno and coworkers, only 7% of patients with SAPHO syndrome presented with both skin and bone changes and in some of them dermatologic manifestations occurred many years before or after the onset of the syndrome [65]. In our material, only one patient had documented skin lesions preceding the diagnosis of SAPHO syndrome.

**Figure 6:**
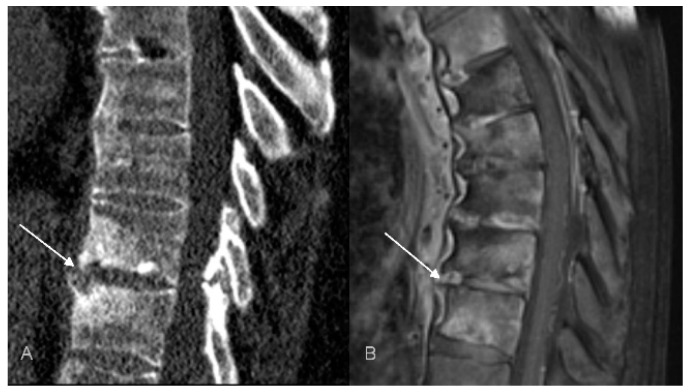
Unstable Anderson lesion in a 65-year-old patient with SAPHO syndrome. Sagittal CT reformat (**A**) and MRI (**B**) shows interruption of syndesmophytes and disc space widening (arrows).

Interestingly, in three patients we have observed the features of pseudoarthrosis in an ankylosed spine (unstable Anderson lesion), a finding that to the best of our knowledge has not yet been reported in patients with CNO/SAPHO syndrome. All pseudoarthroses occurred in the thoracic region and, in one patient, pseudoarthrosis was associated with an aggravation of pain (Figure 6). This observation suggests that in patients with a longstanding CNO and the worsening of symptoms the radiologist should look for a developing pseudoarthrosis, just like in patients with a longstanding AS or with DISH [66].

The main limitation in our study is a relatively small material, especially the underrepresentation of pediatric patients. Since the involvement of costovertebral and facet joints that we found important for the differential diagnosis of CNO has not been previously highlighted, we feel that further studies are needed to confirm the constancy and reliability of this finding. Second, the assessed imaging studies came from five different institutions. However, we found the core elements of imaging protocols sufficiently similar to allow an unbiased assessment.

As has been demonstrated, the findings of prominent BME and osteosclerosis in the vertebral bodies of an adult with a concomitant involvement of spinal joints, but without extensive paraspinal inflammation nor abscess are highly specific for CNO. The diagnosis may be more difficult in children due to a more aggressive course; however, the involvement of costovertebral and facet joints, best appreciated on CT, should suggest that the process is indeed a destructive spondyloarthritis and not malignancy. When the aforementioned imaging features are present, confirming in the next step the involvement of other characteristic sites (ATW) allows for reaching the diagnosis of CNO without resorting to invasive procedures.

In conclusion, a careful inspection of imaging studies may help reduce the number of biopsies in patients with a suspicion of CNO. Going forward, a growing awareness of imaging manifestations of CNO, together with new diagnostic and theranostic strategies currently being researched, including nano-coating of contrast agents and radiotracers and a wider application of hybrid imaging, promise further improvements in the management of patients with this rare condition [67,68,69]. The development of new registries, such as Eurofever (https://www.printo.it/eurofever/index), will certainly expand our understanding of the mechanisms underlying the development and natural history of CNO, and other conditions included in its differential diagnosis.

## Figures and Tables

**Table 1:** A. Bristol Criteria for the diagnosis of CRMO. B. Kahn Criteria for the diagnosis of SAPHO syndrome (from Kahn; American College of Rheumatology 67th Annual Scientific Meeting, October 2003).

**A. Bristol Criteria for the Diagnosis of CRMO**	
The presence of typical clinical findings (bone pain ± localized swelling without significant local or systemic features of inflammation or infection)	
AND	
The presence of typical radiological findings (plain X-ray: showing combination of lytic areas, sclerosis and new bone formation, or preferably STIR MRI: showing bone marrow edema ± bone expansion, lytic areas, and periosteal reaction)	
AND EITHER	
>1 bone (or clavicle alone) without significantly raised CRP (<30 mg/L)	if unifocal disease (other than clavicle), or CRP >30 mg/L, with bone biopsy showing inflammatory changes (plasma cells, osteoclasts, fibrosis, or sclerosis) with no bacterial growth while not on antibiotic therapy
**B. Kahn Critieria for the diagnosis of SAPHO syndrome** **(from Kahn; American College of Rheumatology 67th Annual Scientific Meeting, October 2003)**	
Inclusion criteria	
Bone ± joint involvement associated with PPP and psoriasis vulgaris Bone ± joint involvement associated with severe acne Isolated sterile (a) hyperostosis/osteitis (adults) CRMO (children) Bone ± joint involvement associated with chronic bowel diseases	
Exclusion	
Infectious osteitis Tumoral conditions of bone Non-inflammatory condensing lesions of bone Exception: growth of propionibacterium acnes.	

**Table 2:** Patient characteristics. F—female; M—male; C—cervical, Th—thoracic, L—lumbar; n/a—not applicable. ATW—anterior chest wall, SIJ—sacroiliac joint, * also whole-body MRI.

Patient	Sex [M/F]	Age at the Initial Study [Years]	Imaging Studies Available				Biopsy	Spinal Region Involved	Other Sites of Involvement	Follow Up Duration
			X-ray	CT	MRI	PET/Sc				
1.	F	75	0	1	0	0	-	Th	ATW	n/a
2.	F	65	11	1	6	2	+ (spine)	Th	ATW, SIJ	5y 10mo 6d
3.	M	54	5	0	11	0	-	Th, L-S	-	5y 2mo 16d
4.	F	35	5	2	2	0	+ (spine)	Th	ATW	3y 4mo 23d
5.	F	54	6	4	2	1	-	Th	ATW	15y 11mo 23d
6.	F	56	0	4	0	0	-	Th	ATW, mandible	3y 1mo 14d
7.	M	65	0	1	0	0	-	Th	ATW	n/a
8.	F	68	0	1	0	0	-	Th	ATW	n/a
9.	F	71	0	1	0	0	-	Th	ATW	n/a
10.	M	62	3	1	1	1	+ (sternum)	C, Th	ATW	n/a
11.	F	59	6	2	5	1	+ (spine)	Th	ATW	3y 4mo 14d
12.	F	63	3	2	1	2	-	Th	ATW	4mo 14d
13.	F	39	3	1	2	1	-	L-S	-	1mo 7d
14.	M	61	4	2	6	2	-	Th, L-S	ATW	1y 5mo 16d
15.	F	26	4	0	4	0	-	Th	ATW, SIJ	n/a
16.	M	10	18	4	8	1	+ (spine)	Th	tibia	8mo 14d
17.	F	11	7	1	6 *	1	+ (spine)	Th, L-S	femur, fibulae, ATW	1l 3m 21d

**Table 3:** Distribution of vertebral bone marrow edema (BME) in CRMO and SAPHO syndrome.

Pattern of BME	Patients (Number of Vertebrae per Patient)		
	All	Children	Adults
corner lesion	5 (3)	1 (1)	4 (3.5)
propagating	6 (2)	2 (1)	4 (2.5)
semicircular	3 (5.6)	0	3 (5.6)
diffuse	8 (2.5)	2 (2)	6 (2.6)

**Table 4:** Key imaging features for differential diagnosis of CNO and other spinal diseases [13,38,43]. AS—ankylosing spondylitis. DISH—diffuse idiopathic skeletal hyperostosis. * usually limited.

	Osteosclerosis	BME	Facet Joints and Costovertebral Joints	Paraspinal Inflammation	Abscess
**CNO**	Extensive	Extensive	+	+ *	-
**Infectious spondylitis**	Variable	Extensive	+	+	+
**AS**	Limited to tiny corner lesions	Small to moderate	+	-	-
**DISH**	None	None to small	-	-	-
**Malignancy**	Possible	Rare	-	-	-

## Data Availability

The data supporting the results of this study may be available upon request.

## Abbreviations

ATW	anterior thoracic wall
AS	ankylosing spondylitis
BME	bone marrow edema
CNO	chronic non-bacterial osteomyelitis
CRMO	chronic recurrent multifocal osteomyelitis
CT	computed tomography
DISH	diffuse idiopathic skeletal hyperostosis
18F-FDG PET/CT	F-18 fluorodeoxyglucose-positron emission tomography/computed tomography
LCH	Langerhans cell histiocytosis
MRI	magnetic resonance imaging
SAPHO	synovitis, acne, pustulosis, hyperostosis, osteitis
SPECT/CT	single-photon emission computed tomography
TB	tuberculosis
WBBS	whole-body bone scintigraphy
